# Longitudinal field studies reveal early infection and persistence of influenza A virus in piglets despite the presence of maternally derived antibodies

**DOI:** 10.1186/s13567-019-0655-x

**Published:** 2019-05-22

**Authors:** Pia Ryt-Hansen, Inge Larsen, Charlotte Sonne Kristensen, Jesper Schak Krog, Silke Wacheck, Lars Erik Larsen

**Affiliations:** 1 National Veterinary Institute, Technical University of Denmark, Kemitorvet Building 204, 2800 Kongens Lyngby, Denmark; 2 IDT Biologika GmbH, Am Pharmapark, 06861 Dessau-Rosslau, Germany; 3 SEGES Pig Research Center, Vinkelvej 11, 8620 Kjellerup, Denmark; 4 Dpt. of Veterinary and Animal Sciences, University of Copenhagen, Grønnegårdsvej 2, 1870 Frederiksberg C, Denmark

## Abstract

A longitudinal study was performed in three Danish farrow to grower (30 kilos) herds over a 4-month period to investigate the dynamics and clinical impacts of influenza A virus (IAV) infections. In each herd, four batches consisting of four sows each with five ear-tagged piglets were included. Nasal swabs and/or blood were sampled from the sows and/or the piglets prior to farrowing and at weeks 1, 3, and 5 and at the end of the nursery period. Clinical examinations were performed at each sampling time. The sows and piglets were tested for IAV and IAV antibodies in nasal swabs and blood samples, respectively. The results revealed three enzootically infected herds, where the majority of the pigs were infected during the first 5 weeks after birth. Infected piglets of only 3 days of age were detected in the farrowing unit, where the sows were also shedding virus. In all herds, low to moderate numbers of infected pigs (ranging from 3.6 to 20.7%) were found to be virus positive in nasal swabs at two consecutive sampling times. Furthermore, clinical signs of respiratory disease were associated with IAV detection. The findings of this study documented that IAV can persist in herds and that piglets as young as 3 days can be infected despite the presence of maternally derived antibodies.

**Electronic supplementary material:**

The online version of this article (10.1186/s13567-019-0655-x) contains supplementary material, which is available to authorized users.

## Introduction

Influenza A virus (IAV) is one of the most important viral pathogens in swine herds globally and is considered a significant cofactor in the porcine respiratory disease complex (PRDC) [1, 2]. IAV was first detected in European pigs in the 1970s [3] and has since been related to acute outbreaks of respiratory disease in swine herds that typically resolved within a few weeks [4, 5]. However, in recent years, a number of studies have shown that the dynamics of IAV infections have changed and that IAV can persist in herds. The change is probably a result of the increased herd size that ensures a weekly flow of naive individuals who can maintain the infection [6–12]. IAV is highly prevalent in Danish swine herds, and the results of the national passive surveillance program have revealed that the prevalence of IAV exceeds 45% in the diagnostic samples submitted from pigs with a history of respiratory disease. This makes IAV the most prevalent pathogen found in relation to PRDC in Denmark [13]. H1N1, H1N2 and H3N2 constitute the majority of the circulating IAV subtypes, and each subtype has a significant variety of different lineages with different genetic traits of avian (av), human (hu) or swine (sw) origin [14]. The most prevalent subtype in Denmark is the H1avN2sw, which has the avian-like hemagglutinin (HA) gene and the neuraminidase (NA) gene from the human-like reassortant swine H3N2sw [15]. In 2010, pandemic A(H1N1)pdm09 appeared in Denmark and is now the second most prevalent subtype, constituting 20% of the strains. Furthermore, the internal genes of this strain have been incorporated into more than 80% of the most prevalent strain H1avN2sw [13]. In addition to these dominating enzootic strains, a number of reassortants have been detected, including strains harboring the HA and NA genes from human seasonal flu strains, indicating that human-to-pig transmission takes place [13, 16].

The change in viral dynamics and the increased complexity of the circulating variants pose a challenge for farmers and veterinarians when determining control methods [17]. Thus, there is a great need for studies designed to increase our knowledge of the transmission dynamics and impacts of IAV under field conditions. Few studies have focused on the transmission of IAV early in the farrowing unit [6], as most studies have initiated sampling at an age close to weaning [11, 12] and have been performed as cross-sectional studies [18, 19]. The primary aim of the present study was to determine the prevalence of influenza-positive pigs over time by conducting an observational longitudinal cohort study in three Danish swine herds. A secondary aim was to investigate the association between virus-positive pigs and clinical signs. It is important to investigate the transmission dynamics and the clinical impact in pigs of this age because the pigs are highly susceptible and because this period includes the time when the pigs go from relying on passive immunity to having an active immune response towards IAV. Furthermore, infected piglets at weaning may be the source of the infection in the nursery unit and further downstream.

## Materials and methods

### Ethical statement

This study was carried out in strict accordance with the guidelines of the Good Experimental Practices (GEP) standard adopted by the European Union. All experimental procedures were conducted in accordance with the recommendations given by the National Veterinary Institute of Denmark.

### Selection of target herds

All herds should fulfill the following criteria: Minimum 300 sows, production from farrowing-30 kilos, weekly production system, history of respiratory disease or laboratory confirmation of IAV, no litter equalization of the ear-tagged piglets, no vaccination against IAV in the past year and no startup of vaccination of either sows or piglets against IAV during the study period.

### Screening for IAV in the target herds

Before a herd was included in the study, a screening for IAV was performed by testing nasal swabs from 5 1-week-old piglets, 5 3-week-old piglets, 10 5-week-old weaners and 10 8-week-old weaners by reverse transcription real-time PCR (RT-rtPCR).

### Description of the included herds

#### Herd 1

This herd had approximately 900 sows and a farrowing area divided into six units with no clear sectioning between age groups. The piglets were weaned at 4 weeks of age. When the piglets reached approximately 15 kilos, they were moved to a separate stable until they were sold at 30 kilos. The herd was not included in the Danish SPF-system [20] but was declared free of porcine reproductive and respiratory syndrome virus (PRRSv). The herd had previously tested positive for H1avN2sw and had recurrent problems with respiratory disease. The herd did not use a strict all-in/all-out strategy in any of the units, and no quarantine stability was used for incoming gilts. In the herd, a high degree of litter equalization was used along with nursing sows. Stables were washed between production rounds in the farrowing unit and disinfected using calcium hydroxide.

#### Herd 2

This herd had approximately 900 sows and a farrowing area divided into four units with no clear sectioning between age groups. The piglets were weaned at 4 weeks of age. When the piglets reached approximately 20 kilos, they were moved to a separate stable until they were sold at 30 kilos. The herd had an SPF herd health status, indicating that the herd tested free of infections annually, including *Mycoplasma hyopneumoniae*, *Actinobacillus pleuropneumoniae* serotype, 2, 6, and 12, PRRSv type 1 and 2, *Brachyspira hyodysenteriae*, *Pasteurella multocida*, *Sarcoptes Scabiei* var. *Suis* and *Haematopinus suis*. The herd had recurrent problems with respiratory disease but had never been tested for the presence of IAV. The herd did not use a strict all-in/all-out strategy in any of the units, but a quarantine stable was used for incoming gilts. In the herd, a high degree of litter equalization was used along with nursing sows. Stables were washed between batches and disinfected using calcium hydroxide.

#### Herd 3

This herd had approximately 450 sows and a farrowing area divided into two units with no clear sectioning between age groups. The piglets were weaned at 4 weeks of age and kept in the same grower unit until they were sold at 30 kilos. This herd was known to be IAV-positive and had recurrent clinical signs of respiratory disease. Similar to Herd 2, the herd had a blue SPF status, indicating that it was declared free of the same diseases as Herd 2. The herd only performed minimal litter smoothing and limited the use of nursing sows. Gilts were recruited from the same herd. Stables were cleaned only once a year, without disinfectants.

### Study design

This investigation was designed as an observational longitudinal cohort study in 3 Danish sow herds. In each herd, four batches of four conveniently selected sows were included with farrowing dates 1 week apart. Five piglets from each sow were randomly chosen by ear tagging of every third piglet in the litter at birth. The ear-tagged piglets were sampled with nasal swabs during weeks 1, 3, and 5 and again before being sold at approximately 30 kilos (at 10–12 weeks of age). As the piglets were not born on the same day, the actual sampling date differed up to 4 days between pigs. Furthermore, the ear-tagged pigs were blood sampled during week 3 and at approximately 30 kilos (weeks 10–12). From sows, blood was sampled 2 weeks before farrowing, and a nasal swab was taken 1 week after farrowing (Table 1). A total of 16 sows and 80 piglets were selected for sampling at weeks 1, 3, 5 and 10–12 over a total period of 4 months in each herd. Ear-tagged piglets stayed with their own mother sow until weaning. The sampling size was initially defined based on assumptions on body weight gains and production results, but these indicators were excluded from the final assessment due to inadequate quality of data from the herds.

**Table 1:** **Sampling program for sows and piglets**

	Two weeks before farrowing	Week 1	Week 3	Week 5	Weeks 10–12
Sows	Blood samples	Nasal swabs			
Piglets		Nasal swabs	Blood sample + nasal swabs	Nasal swabs	Blood sample + nasal swabs

### Sampling

Nasal swabs were collected with a small or large sterile cotton swab (Medical Wire, UK) depending on the age of the animal. The swab was inserted and turned 360° in both nostrils of each pig. Afterwards, the swabs were immersed in Sigma Virocult media (Medical Wire, UK) and kept at 2–8 °C for a maximum of 2 days until RNA extraction.

Blood was sampled from *vena jugularis* of the sows and from *vena cava cranialis* of the piglets and stored in vacutainer serum tubes (Becton–Dickinson, Denmark) at 5 °C for a maximum of 2 days until they were centrifuged at 3000 rpm for 10 min, and the serum was frozen at −20° until further analysis.

### Clinical observations

Each individual ear-tagged pig was examined for the presence of the following clinical signs at each sampling time: dyspnea, lacrimation, nasal discharge (s = serous, m = mucous and p = purulent), conjunctivitis, diarrhea and lameness. Additionally, the pigs had a body condition score specified ranging from 1 to 4. Every pen with an ear-tagged piglet had a coughing index (CI) calculated at every sampling time using a method based on a previous study on *Mycoplasma hyopneumoniae* [21]. The CI was calculated based on the number of coughs and sneezes over 3 min divided by the number of pigs in the pen.

### Pooling of the samples and RNA extraction

The nasal swabs were pooled per litter with five samples in one pool corresponding to the five ear-tagged piglets from each sow. Four sows of each batch were also pooled. The Sigma Virocult media containing the cotton swab were vortexed and poured into a 1.5 mL tube (Eppendorf), wherefrom 100 µL was withdrawn for the pool. The pool was vortexed and centrifuged, and 200 µL was withdrawn and mixed with 400 µL RLT-buffer (QIAGEN, Copenhagen, Denmark) containing 2-mercaptoethanol (Merck, Darmstadt, Germany). The RNA was extracted from the sample using the RNeasy mini kit (QIAGEN) automated on the QIAcube (QIAGEN) according to instructions from the supplier.

### Reverse transcription real-time RT-PCR

A previously published RT-rtPCR assay targeting the matrix gene of IAV [22] was used to determine if a pool was IAV positive. The OneStep RT-PCR Kit (QIAGEN) was used with the published primers. All PCRs were run on the Rotor-Gene Q (QIAGEN) using the following program: 50 °C, 30 min; 95 °C, 15 min; and cycling 45× (95 °C for 10 s, 60 °C for 20 s, 64 °C for 1 s, 68 °C for 1 s, 72 °C for 30 s). A pool was considered positive if it had a ct value < 36. If a pool tested positive, the RNA was extracted from the individual samples of the pool using the same method as described above. The RNA was then again subjected to the RT-rtPCR assay described above to determine which individual pigs were positive for IAV.

All positive individual samples with a ct value < 31 were then retested using a multiplex RT-rtPCR assay to determine the influenza A subtype. The QuantiTect Rev transcription kit (QIAGEN) was used with the primers and probes from a previous study [23] with a few primer adjustments, as listed in Table 2. PCR was run on the Rotor-Gene Q (QIAGEN) using the following program: 50 °C for 20 min, 95 °C for 15 min, and cycling 40× (94 °C for 60 s and 60 °C for 90 s).

**Table 2:** **List of adjusted primers and probes used for the RT-PCR multiplex for subtyping**

Assay	Primer/Probe	Sequence (5′-3′)
H1pdm	H1fw2sw-2	GAA GTT CAA GCC GGA AAT AGC A
H1av	H1-av-P	*ROX-*TCT GGT TAC GCA GCW GAT CAG AAA AGC AC*-BHQ2*
H3hu	H3-hu_mink-F	GAT GAT GGA GAA AAC TGC ACA CTA
N2sw	N2-F	GAG TAT GGT GGA CBT CAA AYA G
	N2-R	TTG CGA AAG CTT ATA TAG GCA TGA
	N2-P	*AF532-*CCA TCA GGC CAT GAG CCT GAV CCA TA-*BHQ1*
N2hu	N2hu-P	*AF532-*T[+C]A [+A]CT CYA CAT AAA AGC ACC [+G]-*BHQ1*

“F” indicates the forward primer, “R” indicates the reverse primer, and “P” indicates the probe. Letters in “[ ]” indicate a locked nucleic acid (LNA). The letters in italics indicate the reporter and quencher.

### Serology

All blood samples were tested for antibodies against the NP gene of IAV, which is highly conserved between subtypes, using a commercially available blocking ELISA (IDEXX; Influenza A Ab Test; IDEXX Laboratories, Inc.).

### Descriptive and statistical analysis

The prevalence of influenza was determined at the litter, individual pigs and batch levels. The prevalence of the IAV in the litters of each herd was calculated based on the number of litters that were positive at each sampling time from the total number of litters present at each sampling time. For the individual pigs, the prevalence was also based on the number of individual nasal swabs testing positive for IAV subtracted from the total amount of pigs present at each sampling time. In addition, “total prevalence” was estimated based on the total number of individual pigs testing positive at minimum one sampling time during the entire study period compared to the initial number of pigs at the beginning of the study. Finally, the batch level prevalence of IAV was calculated based on the number of individuals testing positive for IAV compared to the total number of pigs included in the batch.

For each herd, a statistical analysis was performed comparing IAV-positive and IAV-negative individuals at a given age (week 1, week 3, week 5 and weeks 10–12) with the presence of one of the clinical signs registered at the individual level using the Chi-square test. This analysis was also performed on the total clinical data from all three herds to evaluate an overall association. To reveal a possible significant difference between being a litter/pen with at least one IAV-positive animal and being a negative litter/pen in relation to the coughing index, a Fisher’s exact test was performed on the means of the CIs. This test was performed both herdwise and on the total data of all three herds. A *P*-value below 0.05 was considered statistically significant. The statistics and graphs were completed using GraphPad Software [24] and Microsoft Excel.

## Results

### IAV subtypes

At the time of screening, Herd 1 tested positive for H1avN2sw, and this subtype was also found throughout the study period in all positive pigs except one 5-week-old pig, which was infected with an H1avN1 subtype in the nursery. Herd 2 had A(H1N1)pdm09 detected at the time of screening and was later detected in both the farrowing unit and the nursery stable. H1avN2sw was identified at the time of the screening and was the only subtype circulating in Herd 3 in both the farrowing and nursery unit.

### IAV—at the litter level

The results of the tests of the pooled samples, each including a single litter, indicated that the majority of the litters encountered IAV during the study period (Table 3). At week 1, each of the three herds had IAV-positive litters, with Herd 3 standing out, in that half of the tested litters were positive. In week 3, 33–50% of the litters were positive, and at week 5, the percentage of positive litters was 19, 47 and 63% in Herds 1, 2 and 3, respectively. In contrast, only one litter in total was positive at weeks 10–12 (in Herd 2).

**Table 3:** **Percentage of influenza A virus-positive pools from nasal swabs collected from pigs and tested by RT-PCR**

	Herd 1	Herd 2	Herd 3
Positive pools			
Week 1	(4/16) 25%	(3/15) 20%	(8/16) 50%
Week 3	(8/16) 50%	(5/15) 33.3%	(6/16) 37.5%
Week 5	(3/16) 18.7%	(7/15) 46.7%	(10/16) 62.5%
Weeks 10–12	(0/16) 0%	(1/15) 6.6%	(0/16) 0%
Total	(11/16) 69%	(12/15) 80%	(14/16) 87.5%

### IAV—at the individual level

From all the positive pools, the individual samples from the five ear-tagged pigs were tested. The total % of IAV-positive individuals was estimated by summing the number of infected individuals at each sampling time (weeks 1, 3, 5 and 10–12), regardless of the batch (Table 4).

**Table 4:** **Percentage of influenza A virus-positive pigs from nasal swabs collected from pigs and tested by RT-PCR**

	Herd 1	Herd 2	Herd 3
Week 1	17.3% (13/77^a^)	9.2% (6/65^a^)	34.6% (27/78^a^)
Week 3	16.2% (11/68)	15.9% (10/63)	29.5% (23/78)
Week 5	4.8% (3/62)	20.3% (12/59)	36% (28/78)
Week 10–12	0% (0/61)	2% (1/45)	0% (0/76)
Total	34% (26/77)	41.5% (27/65)	69% (54/78)

^a^The total number of piglets present at the beginning of the study deviates from 80 due to mortality between birth and first sampling. Pigs that have been infected twice only count once in the total prevalence.

Due to mortality during the study, the total number of pigs included was less than planned and varied between herds and sampling times (Table 4).

Herd 1 showed the highest prevalence of infected individuals in the farrowing unit, with ~17% of the individuals being infected at weeks 1 and 3. After the piglets were transferred to the nursery unit, a much lower prevalence (4.8%) was observed at week 5, and none of the pigs tested positive at weeks 10–12.

Herd 2 had a relatively low prevalence of IAV at week 1, with only 9.2% of the piglets being infected; however, at week 3, the prevalence increased to 15.9% and peaked in the nursery, with 20.3% of the pigs being infected at week 5. At weeks 10–12, only one pig tested positive for IAV.

Herd 3 had a more constant but high prevalence of IAV over the first three sampling times. Approximately 30% of the individuals were infected at each sampling time, and the highest prevalence (36%) was observed at week 5 after transfer to the nursery unit. Consistent with the finding in Herd 1, all pigs were negative at weeks 10–12.

### IAV—at the batch level

**Figure 1:**
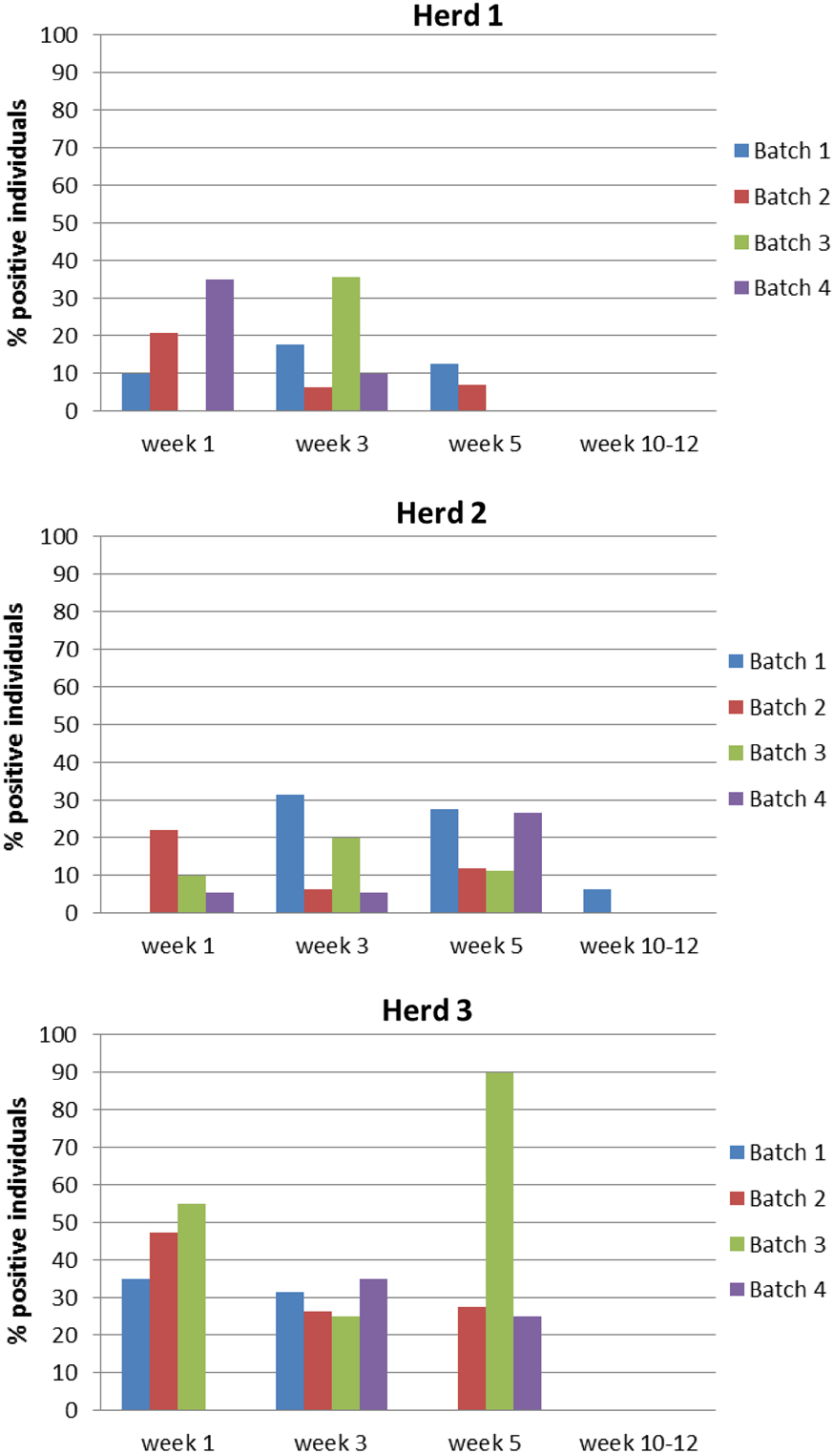
**Percentage of IAV-positive litters in the four batches of the three herds.** The columns present the percentage of positive individuals in each batch at each sampling time (week 1, week 3, week 5 and weeks 10–12) for each of the three herds.

A difference in the time of infection was observed between the different batches, and no clear pattern was observed overall when comparing the three herds (Figure 1). However, in Herd 1 and Herd 3, the batches in which an IAV-positive sow was present, a high number of infected piglets at week 1 were observed (IAV results of the sows are shown below). In Herd 1, the prevalence of infected piglets at week 1 ranged between 21 and 35% in the two batches that had an IAV-positive sow (Batch 2 and Batch 4), whereas the number was even higher in Herd 3, ranging from 47 to 55% of piglets in the two batches including an IAV-positive sow (Batch 2 and Batch 3). In Herd 3, all the piglets of the two IAV-positive sows were infected at week 1, whereas this was the case for one of the IAV-positive sows in Herd 1 (Additional file 1).

### Shedding period and viral load

In all herds, several pigs tested positive for IAV at two consecutive sampling times. In Herd 1, two pigs were positive for IAV in weeks 1 and 3, and in Herd 2, one pig was positive for IAV at both week 3 and week 5. In Herd 3, a much higher prevalence was observed, with eleven pigs testing positive at two consecutive sampling times, and one pig even tested positive over three consecutive samplings (week 1 to week 5). Out of the ten remaining pigs in Herd 3, four were positive for IAV in weeks 1 and 3, while six were positive in weeks 3 and 5. The overall prevalence of infected pigs that were positive over two consecutive samplings ranged from 3.6 to 20.7% of the total number of infected pigs in each herd.

In addition to pigs that were positive at two consecutive samplings, one and 13 piglets were infected at two nonconsecutive sampling times (week 1 and week 5) in Herds 1 and 3, respectively. Detailed results are shown in Additional file 1.

The average ct value of the IAV-positive pigs in Herd 1 increased with age. In contrast, in Herds 2 and 3, the lowest average ct values were detected at week 5, which for both herds coincided with the peak of infected individuals.

### IAV antibodies—ear-tagged pigs

**Figure 2:**
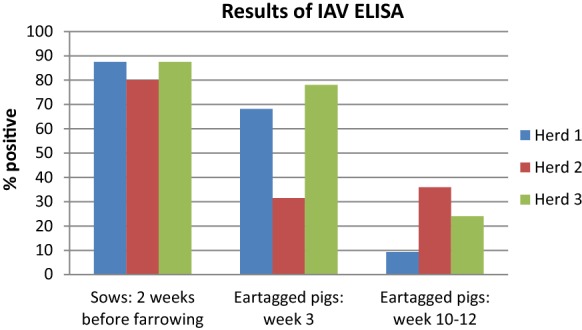
**Prevalence of IAV antibodies at the different sampling times of both sows and piglets of the three herds.** The columns present the proportion of IAV seropositive sows 2 weeks before farrowing and seropositive piglets at weeks 3 and 10–12.

The prevalence of antibodies at weeks 3 and 10–12 of the ear-tagged pigs is shown in Figure 2. Herds 1 and 3 showed a similar pattern, where 68–78% of the piglets were positive for IAV antibodies at week 3, whereas a significant decline was observed at weeks 10–12, where only 9–24% of the piglets were positive. Herd 2 showed a different pattern, with 31–36% of the pigs IAV antibody positive at both sampling times. No clear relationship between virus positivity and serological status was detected, as both piglets originating from antibody-positive sows and pigs from antibody-negative sows became infected during the first 5 weeks after farrowing (Additional file 1).

### IAV and IAV antibodies—sows

The majority (80–87.5%) of all included sows tested positive for antibodies against IAV 2 weeks before farrowing (Figure 2). In Herd 1, two sows were shedding IAV in the farrowing unit at week 1, and one of these sows was antibody negative 2 weeks before farrowing. The exact same pattern was observed in Herd 3, where two sows were also shedding IAV in the farrowing unit, and one tested negative for IAV antibodies 2 weeks before farrowing. In Herd 2, none of the tested sows were virus positive in the farrowing unit (Additional file 1).

### Clinical signs

A statistically significant correlation was identified in Herd 1 between the IAV-positive litters/pens and an increased coughing index compared to the negative litters/pens (Table 5).

**Table 5:** **Mean coughing index (CI) of virus-positive and -negative animals**

Mean CI	Week 1	Week 3	Week 5	Total
Herd 1				
Virus positive	0.209	0.381	0.032	0.263
SD	0.21	0.28	0.04	0.26
Virus negative	0.026	0.223	0.05	0.089
SD	0.03	0.19	0.03	0.13
*P*-value	0.007	0.21	0.44	0.006
Herd 2				
Virus positive	0.042	0.348	0.109	0.168
SD	0.05	0.16	0.08	0.16
Virus negative	0.109	0.56	0.088	0.234
SD	0.12	0.53	0.05	0.36
*P*-value	0.40	0.41	0.48	0.48
Herd 3				
Virus positive	0.065	0.186	0.083	0.108
SD	0.04	0.09	0.03	0.08
Virus negative	0.090	0.239	0.106	0.166
SD	0.07	0.26	0.05	0.2
*P*-value	0.40	0.64	0.45	0.26

The results were considered significant at *P* < 0.05. “SD” is the standard deviation.

An additional statistically significant correlation was identified in Herd 2 between the presence of serous nasal discharge and the individual pig testing positive for IAV in the nasal swabs. This correlation was observed at week 1 and week 5 and equally when looking at the total number of infected pigs (Table 6).

**Table 6:** **Prevalence of nasal discharge of virus-positive and -negative animals**

	Week 1	Week 3	Week 5	Total
Herd 1				
Virus positive	23% (3/13)	30.1% (4/13)	0% (0/3)	36.8% (7/19)
Virus negative	12.5% (8/64)	47.3% (26/55)	50.8% (30/59)	35.9% (64/178)
*P*-value	0.58	0.43	0.26	0.861
Herd 2				
Virus positive	100% (6/6)	70% (7/10)	83.3% (10/12)	82.1% (23/28)
Virus negative	44% (26/59)	32.7% (17/52)	34% (16/47)	37.3% (59/158)
*P*-value	0.03	0.062	0.006	< 0.0001
Herd 3				
Virus positive	77.8% (21/27)	91.3% (21/23)	78.6% (22/28)	82% (64/78)
Virus negative	62.7% (32/51)	81.8% (45/55)	86% (43/50)	76.9% (120/156)
*P*-value	0.26	0.47	0.6	0.46

The results were considered significant at *P* < 0.05.

As 69% of all the individual pigs of Herd 3 were positive for IAV at some point during the study (Table 4), it was not possible to obtain a significant correlation for any of the clinical signs, even though several signs of respiratory disease were observed in the herd.

An overall analysis was performed to investigate whether the above mentioned associations were also apparent when accumulating the results of all three herds (Table 7). The coughing index and nasal discharge for all herds at each of the sampling times and as a total over all sampling times were calculated. A significant correlation between nasal discharge and the individual pig testing positive for IAV in the nasal swabs was observed both at week 1 and week 5 and when looking at the total number of infected pigs. However, no significant correlation was found with regard to the coughing index.

**Table 7:** **Accumulated results of the clinical data from all three herds (CI and nasal discharge)**

Mean CI	Week 1	Week 3	Week 5	Total
Herds 1, 2 and 3				
Virus positive	0.099	0.31	0.092	0.176
SD	0.12	0.22	0.07	0.18
Virus negative	0.072	0.348	0.078	0.17
SD	0.09	0.39	0.05	0.27
*P*-value	0.43	0.70	0.52	0.87
Prevalence of nasal discharge				
Virus positive	65.2% (30/46)	69.6% (32/46)	74.4% (32/43)	69.6% (94/135)
Virus negative	38% (66/174)	55.3% (88/162)	57% (89/156)	49.4% (243/492)
*P*-value	0.002	0.093	0.059	< 0.0001

Results were considered significant at *P* < 0.05. “SD” is the standard deviation.

## Discussion

The results of the present study revealed that IAV was clearly circulating in the farrowing unit, as well as in the start of the nursery period. The majority of the litters encountered IAV at some point during the study, and the true prevalence of IAV-infected individuals was probably higher since the pigs were not sampled every week. To our surprise, IAV was detected at a high prevalence even in piglets at 1 week of age, which to the best of our knowledge has not been described before. Overall, 98% of all the infected pigs tested positive within the first 5 weeks of life, even though more than 80% of the sows were seropositive for antibodies against IAV at farrowing. The high prevalence of seropositive piglets at week 3 (68–78%) in Herd 1 and Herd 3 indicated that the piglets did receive MDAs from the sows. Nevertheless, the results also revealed that these pigs still became infected with IAV at an early age. This can either be due to the level and specificity of the MDAs absorbed by the piglets [25], which was not tested in this study, or due to a lack of protection through MDA, which several studies have indicated [11, 26–31]. Interestingly, the piglets of Herd 2, which did not have a high rate of seropositive pigs at week 3 (31.5%), did not show an overall higher amount of infected individuals. Moreover, Herd 2 had the highest number of seropositive pigs by the end of the study, suggesting that more pigs from this herd have elicited an active immune response to IAV and thereby may be less susceptible to reinfection. This is in accordance with the results from Loeffen et al. [30], who suggested that the presence of MDA in the piglets hindered an active immune response and that these pigs elicited a weaker immune response in response to a secondary IAV infection even with the same subtype.

The differences in the age of infection observed between the batches could be explained by the majority of the batches being in different stables, presumably with different infection pressures, different mixing of age groups and other differences in flow and management factors. Additionally, the presence and prevalence of IAV-positive sows could be a possible factor when considering batch-to-batch variation. In this study, it was observed that three out of the four positive sows also had a positive litter at week 1, which could indicate that the sows affect the transmission dynamics.

Overall, few pigs were infected at the end of the nursery period in three herds, indicating a good chance of having IAV-negative pigs at the time of transfer to the finisher section. However, at weeks 10–12, the prevalence of IAV antibody-positive animals was low, which could indicate that the majority of the pigs would not be protected against IAV reinfection in the finisher farm. Two previous studies have tested this hypothesis and tried to reinfect previously infected piglets with the same strain after the decline in MDA. In one of the studies, the piglets were found to be primed, and no reinfection was possible [29], whereas the other study showed a weakened immune response in the presence of MDA and showed that reinfection was possible in some of the pigs [30]. The decline in IAV antibodies observed at the last sampling time was in accordance with other studies that have found that MDA persists in piglets for approximately 10 weeks [11, 12, 32, 33].

Nonconsecutive shedding of IAV was found in Herd 1 and Herd 3, where piglets were shedding virus at week 1 and again at week 5, with 4 weeks in between. Other studies investigating the IAV dynamics [12, 19] also found pigs that tested positive for the same IAV subtype at nonconsecutive sampling times, which suggested that reinfection with the same virus was possible. An explanation for why we did not see more cases of reinfection could be the detection limit of the PCR assay, which would not detect pigs with a low viral load. The number of positive pigs over a minimum of two consecutive sampling times indicated that individual pigs had viral excretion for more than 2 weeks, which would suggest the presence of “prolonged IAV shedders”. However, as the pigs were not sampled daily, we cannot rule out that these pigs either became reinfected with IAV between the two sampling times or that an environmental contamination of the sample could have occurred. More studies with daily samplings should be performed to prove the concept of prolonged IAV shedders. If we consider that the pigs were in fact true “prolonged IAV shedders”, it is important to take this into consideration in the control measures for IAV, as they will increase the transmission rate. Previous studies have found prolonged shedding time to be correlated to the presence of MDA at the time of infection [26, 27, 30]. This phenomenon should be investigated in more detail because it may be an unwarranted effect of sow vaccination or immunity due to prior infections. A contributing factor to Herd 3 having a much higher prevalence of IAV may be related to the organization of the farrowing unit. Herd 3 only had two large farrowing stables, and so a division into age groups was impossible. Herd 1 and Herd 2, on the other hand, had a higher number of farrowing stables, making it possible to keep the youngest and the oldest pigs more separated, even though a clear sectioning was not possible. This underlines the importance of separation of age groups and strict all-in/all-out strategies when fighting viral pathogens such as IAV [34].

The subtypes found in the three herds represent the subtypes that are currently circulating in Denmark. The most prevalent subtype H1avN2sw was found in Herds 1 and 3, and Herd 1 also had one pig in the nursery unit that tested positive for a different subtype H1avN1av. As none of the pigs in the farrowing unit were infected with this subtype, combined with the low seroprevalence at the end of the nursery period, this different subtype poses a risk of a secondary IAV infection.

Although a descriptive study is not designed to evaluate any associations, the observations regarding influenza and clinical signs were analyzed, and the clinical data showed that there was a significant correlation between being positive for IAV in nasal swabs and clinical signs of respiratory disease. In Herd 1, an increased coughing index was observed. However, this correlation was not observed when accumulating the results of all three herds. In Herd 2, a significant correlation was observed between serous nasal discharge and the presence of IAV, and this correlation was also significant when accumulating the results of all three herds. This indicated that IAV had an impact on health in these enzootically infected herds.

IAV was detected in two out of 16 sows in both Herds 1 and 3, which showed sows as a potential source of exposure of the piglets in the herds and as a possible source of new IAV introductions into the farrowing unit as previously proposed [11, 35]. All piglets from three of the four IAV shedding sows tested positive for IAV at week 1, which could indicate that the virus was transmitted between piglets and sows. However, another explanation could be that both sows and piglets were infected by aerosols and fomites, since the infection pressure in the farrowing unit was quite high. Cases in which both sows and piglets are found positive at the same time need to be further investigated to evaluate the risk of sows transmitting influenza to piglets during the farrowing stage.

In each herd, one of the IAV-positive sows tested negative for IAV antibodies 2 weeks before farrowing, and 1 week after farrowing, these sows were shedding IAV, which indicated that the sows were most likely infected at entry into the farrowing unit, where an abundant circulation of IAV was present. These results emphasize the importance of having a clear introduction strategy of incoming gilts because gilts may either be seronegative if they originate from a non-IAV-infected herd or have antibodies against a different variant of IAV. Exposure to IAV by vaccination before insemination and before farrowing should be considered to reduce the risk of the sows being infected during pregnancy or when entering the farrowing unit. Indeed, gilts have previously been shown to be a contributing factor to IAV persistence at the herd level [18, 36]. Quarantine measures and testing of incoming gilts should be performed to avoid the introduction of new IAV strains into the herd, causing an epizootic outbreak.

All the herds included in the study were enzootically infected with IAV, and signs of clinical impacts were evident. A high infection pressure of IAV was discovered in both the farrowing unit and the start of the nursery period. Interestingly, these results indicated that most of the IAV infections occurred at an age when the piglets were considered clinically protected through MDA. Overall, 98% of all the infected piglets became infected before reaching 6 weeks of age. This indicated that MDAs might not provide optimal protection against IAV, and other control measures, such as improved external and internal biosecurity, should be considered when selecting a strategy for controlling IAV. Finally, sows should be considered highly important players in ongoing IAV transmission and as a possible source of new IAV introductions.

## Additional file

**Additional file 1.** **Overview of the antibody and virus shedding status of the sows and ear-tagged pigs at the different sampling times**. The table shows the four different batches of sows and their respective piglets at the different sampling times. The numbers indicate the ear tag number of the piglets, while the sows are numbered from 1–16. Italic letters indicate that the pig was not blood sampled, bold letters indicate an IAV antibody seropositive ear-tagged pig or sow, and normal letters indicate an IAV antibody seronegative ear-tagged pig or sow. “+IAV” indicates the nasal swab of the individual pigs or sows that tested positive in the quantitative real time RT-PCR targeting the matrix gene of IAV. If a box is empty, it indicates that the ear-tagged pig is either dead or not sampled.

## Abbreviations

Av: avian
HA: hemagglutinin
Hu: human
NA: neuraminidase
IAV: influenza A virus
MDA/MDAs: maternally derived antibody/antibodies
PCR: polymerase chain reaction
Pdm: pandemic
PPRSv: porcine respiratory and reproductive syndrome virus
PRDC: porcine respiratory disease complex
Sw: swine
swIAV: swine influenza A virus
