# Supplementary material for: Longitudinal field studies reveal early infection and persistence of influenza A virus in piglets despite the presence of maternally derived antibodies
Source: Vet Res. 2019 May 22;50:36. doi: 10.1186/s13567-019-0655-x (PMC6530179; doi:10.1186/s13567-019-0655-x)
Supplement: Supplementary file 1 [file 13567_2019_655_MOESM1_ESM.docx]

**Herd 1**	Antibodies and IAV shedding															
	Batch 1				Batch 2				Batch 3				Batch 4			
	**SOW 1**	**SOW 2**	**SOW 3**	**SOW 4**	**SOW 5**	**SOW 6**	**SOW 7**	SOW 8 + IAV	**SOW 9**	**SOW 10**	**SOW 11**	SOW 12	**SOW 13**	**SOW 14**	**SOW 15 + IAV**	**SOW 16**
Week 1	11	20	6	2 + IAV	22 + IAV	29	36	41	43	51	55	66	75	68	58 + IAV	80 + IAV
	14	8	12	16 + IAV	25 + IAV	30	32	39	42	49	56	63	72	71	59 + IAV	81
	19	5	17	18	23	31	35	40	45	50	52	64	76	67	57 + IAV	77
	15	4	13	1	24 + IAV	27	34	38	46	48	53		74	69	61 + IAV	78 + IAV
	3	10	9	7	21 + IAV	28		37	44	47	54		73	70	60 + IAV	79
Week 3	**11**	20	**6**	2	**22 + IAV**	**29**	**36**	41	43	**51**			**75**	**68**	58	**80**
	**14**	8 + IAV	**12**	16	**25**	30	**32**	39	**42 + IAV**	**49**		63 + IAV	**72**	71	**59**	81
	**19**	5	**17 + IAV**		**23**	31	**35**	40	**45**	**50 + IAV**	52	64 + IAV	76	67	57 + IAV	**77 + IAV**
	15	4	13		**24**	27	34		**46**	**48**			**74**	**69**	**61**	78
	**3**	10 + IAV	**9**		21				**44 + IAV**	**47**	54		73	70	**60**	79
Week 5	11	20	6	2 + IAV		29	36	41	43				75	68		80
	14	8		16	25	30	32	39	42	49		63	72	71	59	81
	19 + IAV	5	17		23	31	35		45	50	52	64	76	67	57	77
	15	4	13		24		34		46	48			74	69	61	78
	3	10	9		21	28 + IAV			44	47	54		73	70	60	79
Week 10-12	11	**20**	6	2	22	**29**	36	41	43				75	68		80
	14	8		16	25	30	32	39	42	49		63	72	71	59	81
	**19**	**5**	17		23	31	35		45	50	52	64	76	67	57	
	15	4	13		24	27	34		46	48			74	69	61	78
	3	10	9		21	28		37	44	47			73	70		79

**Herd 2**	Antibodies and IAV shedding															
	Batch 1				Batch 2				Batch 3				Batch 4			
	SOW 1	**SOW 2**	**SOW 3**	**SOW 4**	**SOW 5**	**SOW 6**	**SOW 7**	SOW 8	**SOW 9**	**SOW 10**	SOW 11	**SOW 12**	**SOW 13**	**SOW 14**	**SOW 15**	**SOW 16**
Week 1	105	111	127	106	145	121	118	134	151	143	140 + IAV		144	164 + IAV	175	170
	102	113	129	107	137	123	117	131 + IAV	152	150			158	162	166	171
	104	115	128	109	138	120	119	136 + IAV	153	142			157	163	167	174
	101	112	126	108	139	122	116	133 + IAV	154	141			159	161	176	173
	103	114	130		135			132 + IAV	155				156			172
Week 3	105 + IAV	111	**127**	106	145	**121**	118	134	151	143	140		144	**164**	175	**170**
	102 + IAV	**113**	129	107	137	**123**	117	**131**	152	150 + IAV			158	162 + IAV	**166**	171
	104 + IAV	115 + IAV	**128**	109		120	119	136	153	142 + IAV			157	**163**	**167**	174
	101 + IAV	112	126	108	139	**122**		133	**154**	141			159	161	176	**173**
	**103 + IAV**	**114**	130		135 + IAV			132	155				156			**172**
Week 5	105	111	127 + IAV		145 + IAV	121	118	134		143	140		144	164	175	
	102	113	129	107	137	123	117	131	152	150			158	162		171
	104	115	128 + IAV	109 + IAV	138	120	119	136	153	142			157 + IAV	163 + IAV	167	174
	101	112	126 + IAV	108	139	122		133	154 + IAV	141			159	161	176	173 + IAV
	103	114	130 + IAV		135 + IAV			132	155							172 + IAV
Week 10-12	105	111	127	**106**	**145**	121	118		151	143	140			**164**		
	**102**		129 + IAV	**107**	137	**123**			152					162		
	104	115	128	**109**	**138**	**120**	**119**	**136**	153	142				**163**		174
	101	112		**108**	139	**122**		133	**154**	**141**			159		176	
	103		130					132	155				156			

**Herd 3**	Antibodies and IAV shedding															
	Batch 1				Batch 2				Batch 3				Batch 4			
	**SOW 1**	**SOW 2**	**SOW 3**	**SOW 4**	SOW 5 + IAV	**SOW 6**	**SOW 7**	**SOW 8**	**SOW 9**	**SOW 10**	**SOW 11**	**SOW 12 + IAV**	SOW 13	**SOW 14**	**SOW 15**	**SOW 16**
Week 1	401		411 + IAV	416	421 + IAV	426	431 + IAV	436	441	446 + IAV	451	456 + IAV	461	466	471	476
	402 + IAV	407	412 + IAV	417	422 + IAV	427	432 + IAV	437	442	447 + IAV	452	457 + IAV	462	467	472	477
	403 + IAV	408	413 + IAV	418		428	433 + IAV	438 + IAV	443	448 + IAV	453 + IAV	458 + IAV	463	468	473	478
	404	409	414 + IAV	419	424 + IAV	429	434	439	444	449 + IAV	454	459 + IAV	464	469	474	479
	405	410	415 + IAV	420	425 + IAV	430	435 + IAV	440	445	450 + IAV	455	460 + IAV	465	470	475	480
Week 3	**401 + IAV**		**411**	**416**	421	**426**	431	436 + IAV	441	**446**	**451 + IAV**	**456**	461	**466**	**471 + IAV**	476
	**402 + IAV**	**407**	412	**417**	422	**427**	**432**	**437 + IAV**	442	**447**	**452 + IAV**	**457**	**462**	**467 + IAV**	**472 + IAV**	477
	**403 + IAV**	**408**	**413**	418		**428**	**433**	**438 + IAV**	443	**448**	**453 + IAV**	**458**	463	**468 + IAV**	**473 + IAV**	478
	**404 + IAV**	**409**	**414**	**419**	424	**429**	434	439 + IAV	444	**449**	**454 + IAV**	**459**	**464**	**469**	**474 + IAV**	479
	**405 + IAV**	**410 + IAV**	**415**	**420**	**425**	**430**	**435**	440 + IAV	445	**450**	**455 + IAV**	**460**	465	**470 + IAV**	475	480
Week 5	401		411	416	421 + IAV	426	431	436 + IAV	441 + IAV	446 + IAV	451	456 + IAV	461	466	471 + IAV	476
	402	407	412	417	422 + IAV	427 + IAV	432	437	442 + IAV	447 + IAV	452 + IAV	457 + IAV	462	467	472 + IAV	477
	403	408	413	418		428	433	438	443 + IAV	448 + IAV	453 + IAV	458 + IAV	463	468	473 + IAV	478
	404	409	414	419	424	429	434 + IAV	439	444 + IAV	449 + IAV	454 + IAV	459 + IAV	464	469	474 + IAV	479
	405	410	415	420	425	430	435		445 + IAV	450 + IAV	455	460 + IAV	465	470	475	480 + IAV
Week 10-12	**401**		**411**	416	**421**	426	431	**436**	441	446	451	456	**461**	466	471	476
	**402**	**407**	412	417	**422**	427	432	437	442	447	452	457	462	467	472	477
		**408**	**413**	418		428	433	438	443	448	453	458	463	468	473	478
	404	**409**	**414**	**419**	424	429	434	439	444	449	454	459	**464**	469	474	479
	**405**	**410**	415	**420**	**425**	430	435		445	450	455	460	465	470	475	**480**
